# The negative charge of the 343 site is essential for maintaining physiological functions of CXCR4

**DOI:** 10.1186/s12860-021-00347-9

**Published:** 2021-01-23

**Authors:** Liqing Wang, Qiuhong Xiong, Ping Li, Guangxin Chen, Nayab Tariq, Changxin Wu

**Affiliations:** 1 Institutes of Biomedical Sciences, Shanxi University, Taiyuan, 030006 China; 2 Key laboratory of Medical Molecular Biology of Shanxi Province, Shanxi University, Taiyuan, 030006 China

**Keywords:** WHIM, CXCR4, Mutation, Residue charge changing, Abnormal activities of signal pathway

## Abstract

**Background:**

Warts, hypogammaglobulinemia, recurrent bacterial infections and myelokathexis (WHIM) syndrome is a primary immunodeficiency disease (PID) usually caused by autosomal dominant mutations in the chemokine receptor *CXCR4* gene. To date, a total of nine different mutations including eight truncation mutations and one missense mutation (E343K, CXCR4^E343K^) distributed in the C-terminus of CXCR4 have been identified in humans. Studies have clarified that the loss of phosphorylation sites in the C-terminus of truncated CXCR4 impairs the desensitization process, enhances the activation of G-protein, prolongs downstream signaling pathways and introduces over immune responses, thereby causing WHIM syndrome. So far, there is only one reported case of WHIM syndrome with a missense mutation, CXCR4^E343K^, which has a full length of C-terminus with entire phosphorylation sites, no change in all potential phosphorylation sites. The mechanism of the missense mutation (CXCR4^E343K^) causing WHIM syndrome is unknown. This study aimed to characterize the effect of mutation at the 343 site of CXCR4 causing the replacement of arginine/E with glutamic acid/K on the receptor signal transduction, and elucidate the mechanism underling CXCR4^E343K^ causing WHIM in the reported family.

**Results:**

We completed a series of mutagenesis to generate different mutations at the 343 site of CXCR4 tail, and established a series of HeLa cell lines stably expressing CXCR4^WT^ or CXCR4^E343D^ (glutamic acid/E replaced with aspartic acid/D) or CXCR4^E343K^ (glutamic acid/E replaced with lysine/K) or CXCR4^E343R^ (glutamic acid/E replaced with arginine/R) or CXCR4^E343A^ (glutamic acid/E replaced with alanine/A) and then systematically analyzed functions of the CXCR4 mutants above. Results showed that the cells overexpressing of CXCR4^E343D^ had no functional changes with comparison that of wild type CXCR4. However, the cells overexpressing of CXCR4^E343K^ or CXCR4^E343R^ or CXCR4^E343A^ had enhanced cell migration, prolonged the phosphorylation of ERK1/2, p38, JNK1/2/3, aggravated activation of PI3K/AKT/NF-κB signal pathway, introduced higher expression of TNFa and IL6, suggesting over immune response occurred in CXCR4 mutants with charge change at the 343 site of receptor tail, as a result, causing WHIM syndrome. Biochemical analysis of those mutations at the 343 site of CXCR4 above shows that CXCR4 mutants with no matter positive or neutral charge have aberrant signal pathways downstream of activated mutated CXCR4, only CXVR4 with negative charge residues at the site shows normal signal pathway post activation with stromal-derived factor (SDF1, also known as CXCL12).

**Conclusion:**

Taken together, our results demonstrated that the negative charge at the 343 site of CXCR4 plays an essential role in regulating the down-stream signal transduction of CXCR4 for physiological events, and residue charge changes, no matter positive or neutral introduce aberrant activities and functions of CXCR4, thus consequently lead to WHIM syndrome.

**Supplementary Information:**

The online version contains supplementary material available at 10.1186/s12860-021-00347-9.

## Background

Warts, hypogammaglobulinemia, recurrent bacterial infections and myelokathexis (WHIM) is a rare autosomal dominant inherited primary immunodeficiency disease (PID) [1–3]. Patients with WHIM syndrome with low concentration of B cells in the blood are susceptive to human papillomavirus (HPV) infection, and get warts which is difficult to control using standard medical treatment [4, 5]. Recurrent bacterial infection is another clinical feature in WHIM syndrome, which would cause lots of other diseases, including chronic lung disease, joint, hearing dysfunction, loss of dentition [6] and tetralogy of Fallot which is a congenital heart disease [7]. Myelokathexis is another clinical feature of WHIM syndrome. The mature neutrophils retained in the patient’s bone marrow and undergo apoptotic death, therefore resulted in the lack of neutrophils in peripheral stream [8, 9]. In addition to these classical features, the clinical onset and complications of WHIM syndrome are more than initially suspected [10].

**Fig. 1:**
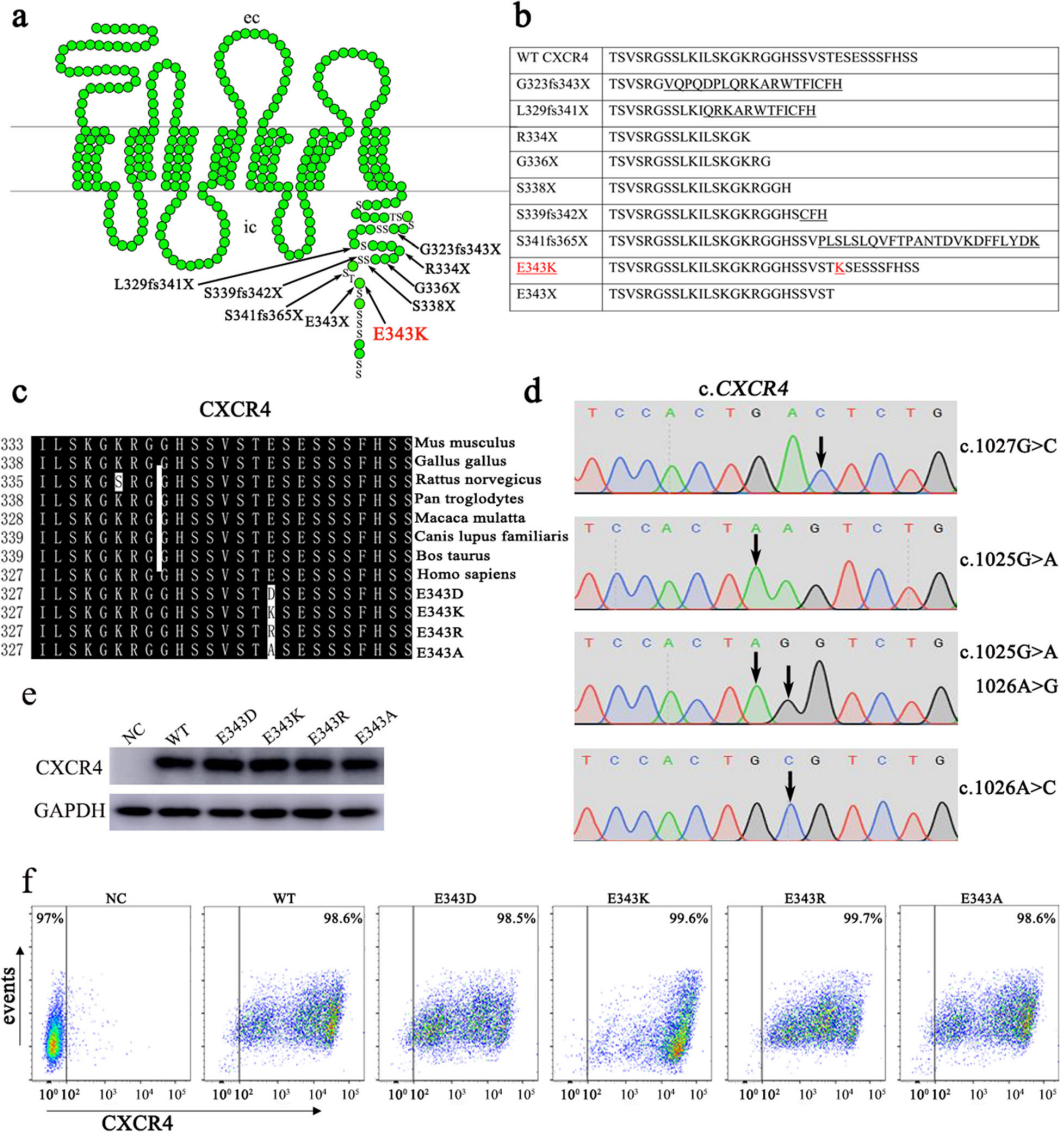
Schematic diagram of mutations in CXCR4. **a** 9 different mutations in the C-terminus of CXCR4 cause WHIM syndrome. **b** The protein sequence of mutant CXCR4. **c** The glutamic acid at 343 site of CXCR4 is highly conserved among species. **d** the mutant CXCR4 were generated by mutagenesis and the vector was confirmed by sequencing. **e** The stably overexpressing wildtype and mutant CXCR4 in HeLa cells were validated by western blot. **f** Flow cytometric analysis of GFP expression in control (negative control, NC) Hela cells and HeLa cells stably transduced with CXCR4 (WT, E343D, E343K, E343R and E343A)

It is reported that almost all cases of WHIM syndrome are caused by mutations distributed in the cytoplasmic C-terminus of cysteine-X-cysteine chemokine receptor 4 (*CXCR4*), which contains eighteen potential serine/threonine phosphorylation sites [11], and six of them are phosphorylated by various kinases such as G protein-coupled receptor kinases (GRKs) and protein kinase C (PKC) upon stimulation with the ligand stromal-derived factor (SDF1, also known as CXCL12). The truncated CXCR4 lost phosphorylation sites impairs the desensitization process, thereby enhancing the activation of G-protein, and causing WHIM syndrome (Fig. 1a) [2, 12]. To date, a total of nine different mutations of 65 cases of WHIM syndrome in humans had been reported by the year 2019 (reviewed by Lauren E. Heusinkveld et al.) [13]. Analysis of those nine WHIM syndrome like mutations shows that almost all result in premature truncated proteins, leading to a loss of serine and threonine residues at the C-terminus of CXCR4, including G323fs343X, L329fs341X, R334X, G336X, S338X, S339fs342X, S341fs365X and E343X, only one mutation association with a single case of WHIM syndrome family from North Carolina causes one residue change from E (glutamic acid) to K (lysine) (CXCR4^E343K^) at the 343 site of the CXCR4 tail, but no loss of serine and threonine residues for phosphorylation (Fig. 1a, b) [2, 14, 15]. The family with CXCR4^E343K^ had clinical manifestations of WHIM syndrome, and experiments showed that the desensitization of activated CXCR4^E343K^ was delayed, cellular calcium flux and cell migration were increased [15], demonstrating in vitro CXCR4^E343K^ is a causative factor for the WHIM syndrome. Fine biochemical analysis shows that the glutamic acid at the site 343 is conserved among all species (Fig. 1c), the mutation causes a positive charge residue of lysine substitution for a negative charge residue of glutamic acid, by which we speculated that the negative charge of the residue is crucial for physiological functions of CXCR4, otherwise, positive or neutral charge residue at the site may cause aberrant activities or functions of the receptor, leading to pathogenic events and eventually causing WHIM syndrome, to verify if our speculation is scientific, a series of experiments of extensive mutagenesis and cell-based functional analyses of the receptors with E343K mutation and other artificially created mutations at the site 343 of the receptor should to be performed and the mechanism underlying the family with CXCR4^E343K^ can be clarify.

In this study, we focused on the E343K mutation in CXCR4, where, we performed a series of mutagenesis, artificially generated a series of CXCR4 mutations at the 343 site and established a series of HeLa cell lines individually stably overexpressing CXCR4^WT^ or CXCR4^E343D^ or CXCR4^E343K^ or CXCR4^E343R^ or CXCR4^E343A^. Functional analyses showed that the cells overexpressing of CXCR4^E343D^ did not accelerate the cell migration and neither enhance the inflammatory responses compared with the cells overexpressing CXCR4^WT^. However, the cells overexpressing of CXCR4^E343K^ increased the cell migration, prolonged the phosphorylation of ERK1/2、p38、JNK1/2/3 and the activation of PI3K/AKT/NF-κB signal pathway, introduced aggravated inflammation responses. Remarkably, overexpressing of CXCR4^E343R^ as well as CXCR4^E343A^ showed the same effect on cells with that overexpressing CXCR4^E343K^. Taken together, all the results suggested that the negative charge at the 343 site is essential for the normal function of CXCR4. The E343K mutation impairs the desensitization and the down-stream signal transduction of CXCR4, consequently lead to WHIM syndrome.

## Results

### Establishment of stably overexpressing HeLa cell lines

To date, only the E343K mutation causing WHIM syndrome has been identified in humans [15]. Therefore, we hypothesized that the negative charge is important for its functions. As HeLa cell does not normally express native CXCR4 on the cell surface what makes HeLa cell such a proper cellular model for studying the function of CXCR4. To confirm our hypothesis, we replaced the Glutamic acid/D with Aspartic acid/E (another acidic amino acid, negative charge E343D), or Arginine /R (another basic amino acid, positive charge, E343R), or a neutral amino acid Alanine/A (E343A) using site-directed mutagenesis technique (Fig. 1d), and established HeLa cell lines that stably overexpressing CXCR4^WT^ or CXCR4^E343K^ or CXCR4^E343D^ or CXCR4^E343R^ or CXCR4^E343A^ respectively to study whether change of the charge at the site 343 affects the functions of CXCR4. Western blot analysis revealed that HeLa cells expressed similar levels of WT or mutant CXCR4 proteins (Fig. 1e). Furthermore, the percentage of cells expressing CXCR4 was more than 98% for HeLa cells stably transduced with CXCR4 wild type and mutants (WT, E343D, E343K, E343R and E343A), and those either stable lines expressing wild type receptor or mutants were maintained at this expression precentage under selective pressure (Fig. 1f).

### Charge-change of the 343 site of CXCR4 promotes cell migration

**Fig. 2:**
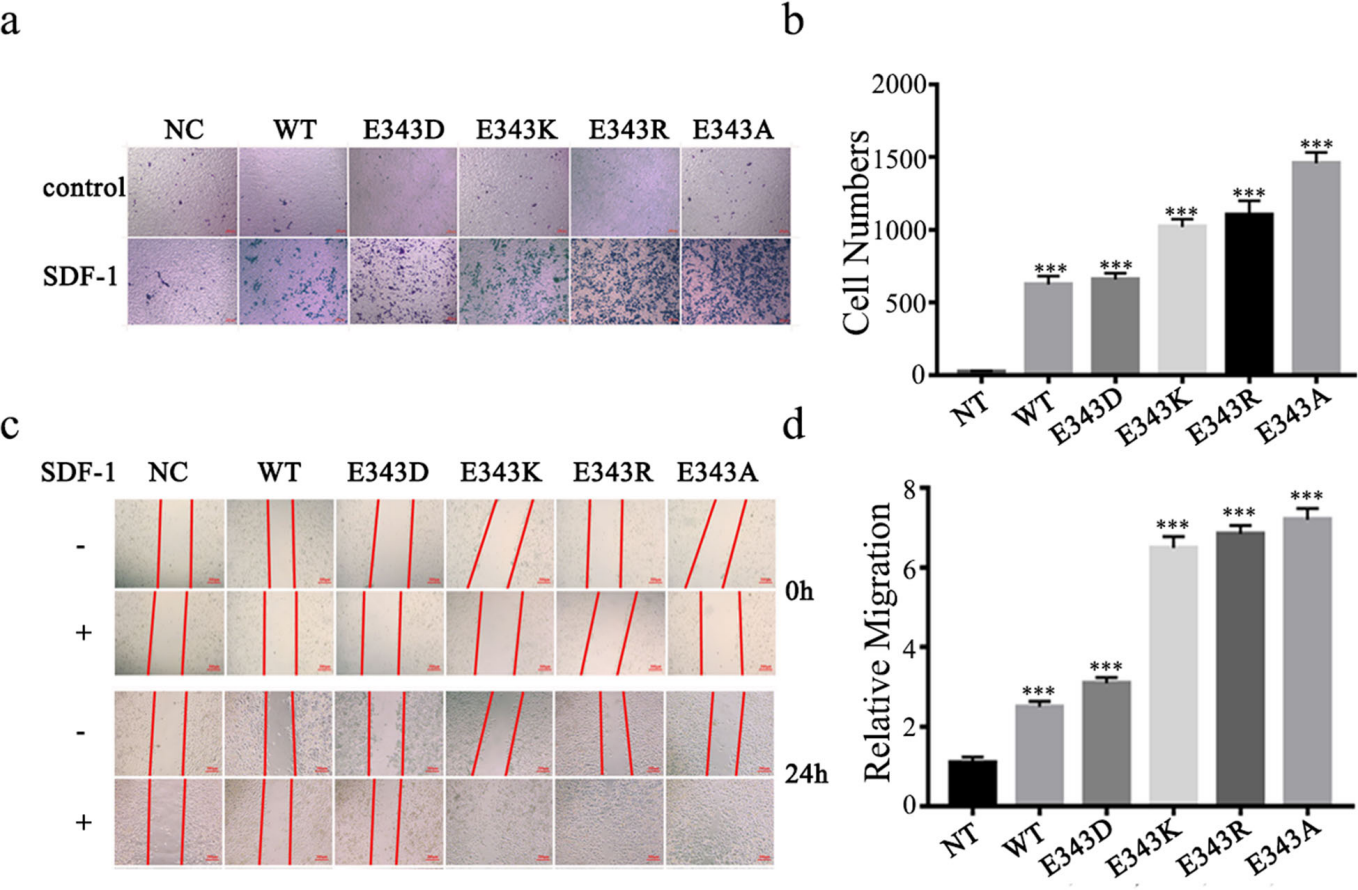
The effect of E343 mutation on cell migration. Cell migration was evaluated by Transwell assay, and the cells migrated through the membrane were fixed in formaldehyde and stained with crystal violet, then imaged using the microscope (**a**) and quantified the cell number using ImageJ software (**b**). Cell migration was evaluated by wound healing assay (**c**) and the migration rate was quantified (**d**). Scale bar: 100 μm, *p < 0 .05, **p < 0 .01, ***p < 0 .001

To study the effect of mutant CXCR4 on the cell migration activities, we detected cell migration using Transwell co-culture system and wound healing assay. The results showed that the wound closure rate and migration was similar between the cells overexpressing CXCR4^WT^ and CXCR4^E343D^ (Fig. 2a-d). However, the migration rate of the cells overexpressing CXCR4^E343K^ was accelerated (Fig. 2a-d), intriguingly cell lines overexpressing CXCR4^E343R^ as well as CXCR4^E343A^ also had enhanced the cell migration, like CXCR4^E343K^ mutant (Fig. 2a-d). These results suggest that the negative charge of 343 site of CXCR4 in important for mentioning normal cell migration activities.

### Charge-change of the 343 site of CXCR4 prolongs the phosphorylation of ERK1/2, p38 and JNK1/2/3

**Fig. 3:**
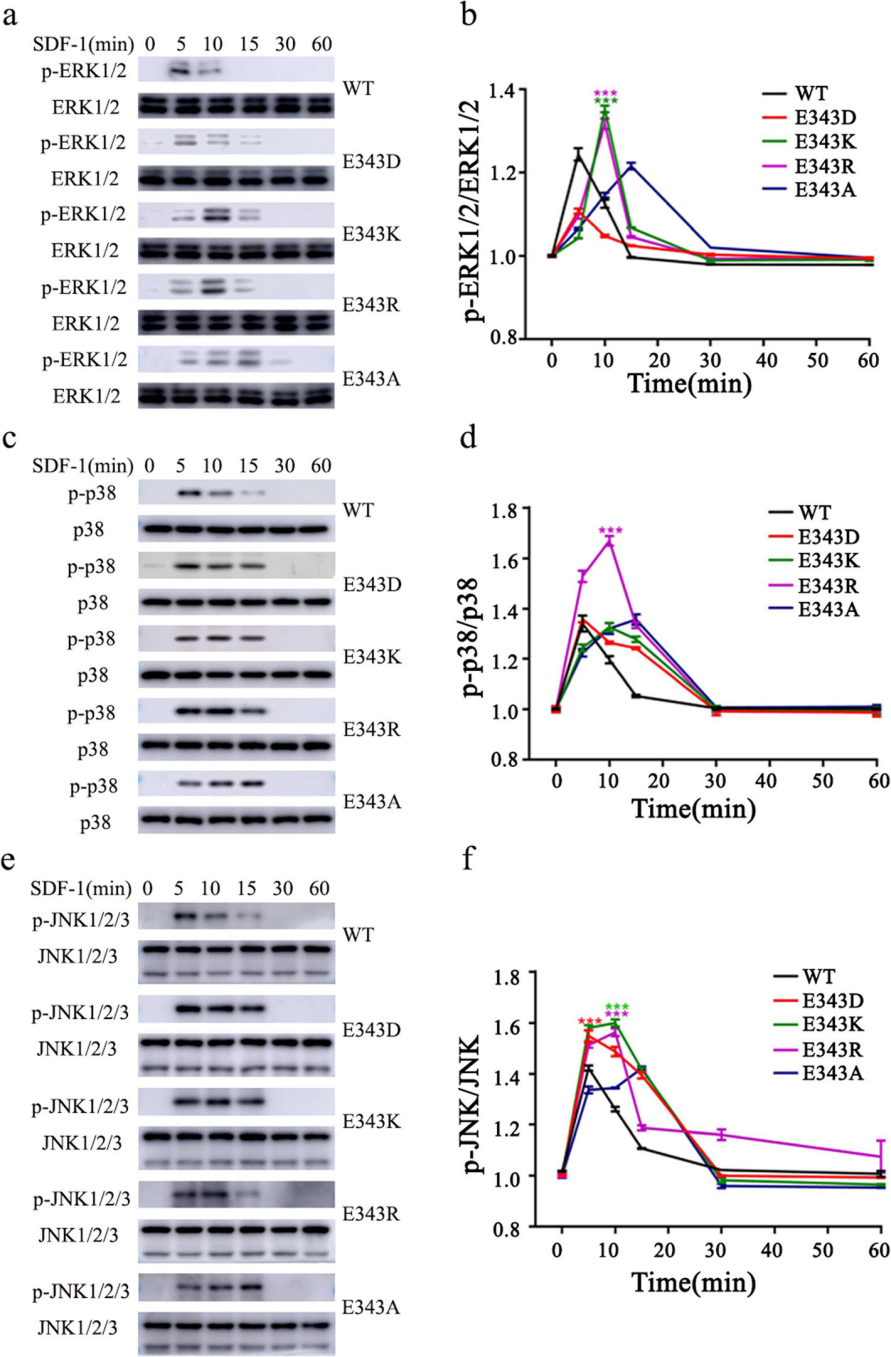
The effect of E343 mutation on MAPK signaling. The stably overexpressing HeLa cells were serum-starved and then exposed to SDF-1 (50 nM) and over a range of time periods (5 min to 60 min). Western blotting was performed for phosphorylated ERK1/2 (**a**), p38 (**c**) and JNK1/2/3 (**e**). Total ERK1/2, p38 and JNK were used as loading control. The relative phosphorylation level was quantified using ImageJ software and showed in (**b**) for ERK1/2, (**d**) for p38 and (**f**) for JNK1/2/3, respectively. Bars represent the mean value ± standard deviation of at least three independent experiments. * *P* < 0.05; ** *P* < 0.01; *** *P* < 0.001

It has been reported that SDF-1 activates CXCR4 and then induces cell migration via ERK, p38 and JNK MAPK pathway [16, 17]. Next, we explored the effect of charge-change of the residue at the 343 site of CXCR4 on MAPK pathway. We detected the phosphorylation of MAPK at different time points post treatment with SDF-1, and found that phospho-ERK1/2, phospho-p38 and phospho-JNK proteins increased significantly in a time-dependent manner (Fig. 3a-f). At 5 min post-treatment with SDF-1, the phosphorylation rate of MAPKs including ERK1/2, p38 and JNK1/2/3 reached a peak, and phospho-ERK1/2, phosphor-p38 and phosphor-JNK1/2/3 were dephosphorylated at 15 min, 30 min and 60 min post-treatment with SDF-1, respectively (Fig. 3a-f). The phosphorylation and dephosphorylation patterns were similar between the cells overexpressing CXCR4^E343D^ and CXCR4^WT^, while the phosphorylation rate reached the peak at 10–15 min post-treatment in cells overexpressing CXCR4^E343K^ or CXCR4^E343R^ or CXCR4^E343A^ (Fig. 3a-f). Consistent with our the results in Supplement Fig. 1 which showed delayed MAPK phosphorylation in mutant CXCR4 receptors compared to that in CXCR4 WT, we found that ligand-induced receptor downstream regulation was delayed in HeLa cells expressing the mutant CXCR4 (E343K or E343R or E343A) compared to HeLa cells expressing the WT receptor (Fig. S1). These results indicate that the charge-change at the 343 site of CXCR4 receptor introduces delayed internalization, prolongs the phosphorylation of MAPKs, prolongates the down-stream signal transduction.

### Charge-change of the 343 site of CXCR4 results in an exacerbation of inflammation response

**Fig. 4:**
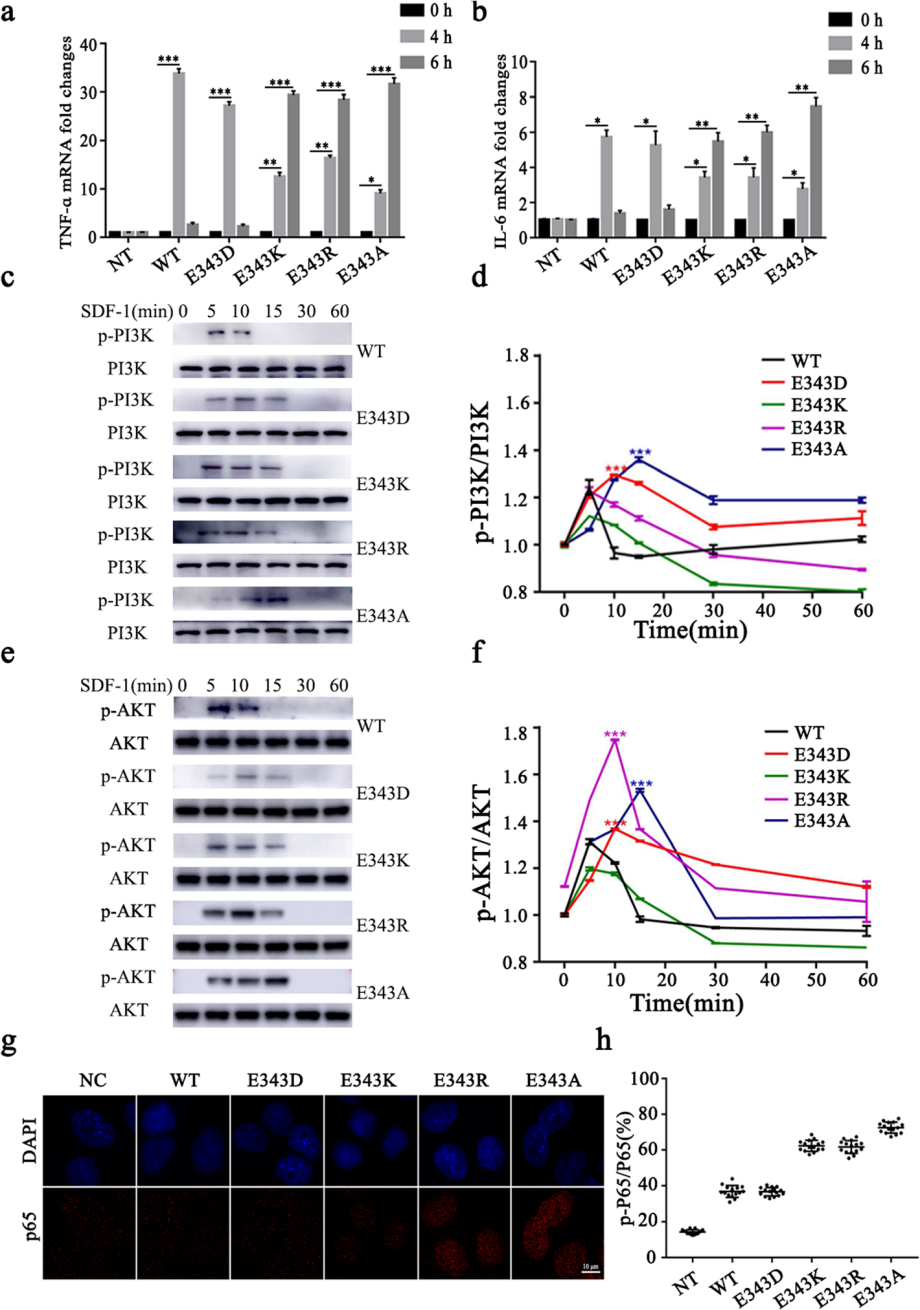
The effect of E343 mutation on inflammatory response. HeLa Cells were stimulated with SDF-1 (50 nM) for 0, 4 and 6 h, respectively. The mRNA expression level of TNF-α (**a**) and IL-6 (**b**) was quantified by qRT-PCR. Western blotting was performed for phosphorylated PI3K (**c**) and AKT (**e**). Total PI3K and AKT were used as loading control. The relative phosphorylation level was quantified using Image J software and showed in (**d**) for PI3K and (**f**) for AKT, respectively. The subcellular localization of p65 was photographed using confocal microscope (**g**) and relative phosphorylated p65 which localized in the nucleus were quantified using ImageJ software (**h**). Bars represent the mean value ± standard deviation (SD). * *P* < 0.05; ** *P* < 0.01; *** *P* < 0.001

Accumulating evidence demonstrated that SDF-1/CXCR4 is involved in inflammatory responses [18–20], knockdown of CXCR4 suppressed the activation of NF-κB signaling pathway therefore prevented the expression of inflammatory cytokines [21]. To determine whether the mutations above impair the inflammatory response of the cell expressing charge changed CXCR4 mutants, we detected the mRNA expression of pro-inflammatory cytokines, TNF-α and IL-6. The results showed that the mRNA expression of TNF-α and IL-6 was dramatically increased in cells overexpressing CXCR4^WT^ or CXCR4^E343D^ at 4 h post-treatment with SDF-1 (Fig. 4a, b), and the mRNA expression returned to basal level at 6 h post-treatment with SDF1 (Fig. 4a, b). However, the mRNA expression was slowly increased and reached the peak at 6 h post- treatment with SDF-1 in cells overexpressing CXCR4^E343K^ or CXCR4^E343R^ or CXCR4^E343A^ (Fig. 4a, b). As PI3K/AKT/ NF-κB signal pathway plays a critical role in the regulation of the various inflammatory cytokines expression [22]. Next, we detected the phosphorylation of PI3K and AKT using western blot, and found that PI3K and AKT were highly phosphorylated at 5 min post-treatment with SDF-1 in cells overexpressing CXCR4^WT^ and CXCR4^E343D^, while PI3K and AKT were highly phosphorylated at 10–15 min in the cells overexpressing CXCR4^E343K^ or CXCR4^E343R^ or CXCR4^E343A^ (Fig. 4c-f). Transcriptional activation of inflammatory cytokines required the nuclear translocation of the NF-κB subunit p65 [23]. Therefore, we examined the localization of p65 after SDF-1 treatment in HeLa cells by immunofluorescence. Consistent with the mRNA expression of pro-inflammatory cytokines, the immunofluorescence results revealed that after stimulation with SDF-1, the nuclear relocalization of p65 lasted until 6 h post-stimulation in the cells overexpressing CXCR4^E343K^ or CXCR4^E343R^ or CXCR4^E343A^, while that was almost diminished in the cells overexpressing of CXCR4^WT^ and CXCR4^E343D^, suggesting the nuclear relocalization of p65 lasted much shorter (Fig. 4g, h). Taken together, these results indicate that the inflammatory response prolonged and enhanced by a charge-changing amino acid substitution in the 343 site of CXCR4.

## Discussion

In the present study, we created more extensive point mutations at the 343 site and completed a series of cell-based functional assays of the mutational receptors in HeLa cells. In particular, we found that the negative charge of amino acid at the 343 site (eg, Glu, Asp) is essential for the normal function of CXCR4, thus mentioning physiological events related with CXCR4. The charge-change substitution mutations (eg, positive residues of Lys and Arg or neutral residue of Ala) delay the desensitization and prolong down-stream signal transduction of CXCR4, and increase cell migration activities and aggravate inflammatory responses, thus causing pathogenic events, consequently leading to WHIM syndrome.

Up to now, 105 cases of WHIM syndrome have been described all over the world, and almost all of them were caused by different mutations causing truncation of the C-terminus of CXCR4, losing phosphorylation sites, but only one missense mutation in CXCR4, and all potential phosphorylation are intact [13, 24–28]. As a G-protein-coupled receptor (GPCR), the down-stream signal transduction is mediated by coupling to an intracellular heterotrimeric G-protein. Studies have demonstrated that the C-terminus of CXCR4 is important for interaction with GRK-β-arrestins which regulate the desensitization and internalization of CXCR4 [29]. Orsini et al. found that phosphorylation of S338/339 were essential for homologous desensitization [30–32], the mutation of CXCR4^E343K^ found in a family with WHIM syndrome does not introduce a loss of any single phosphorylation site in the C-terminus of CXCR4, but the mutation only is close to phosphorylation residues at S346/347 and S338/339. Biochemical analysis shows that the significant change of residue in CXCR4^E343K^ is charge change, a substitution of the negative charge residues with positive residue (Glu/E to Lys/K), by which we made a speculation that the charge change alters the phosphorylation activities of the residues at S346/347 and S338/339 nearby, thus we created a series of mutations which cause different substitutions of the residue at 343 site of CXCR4 C-terminus, and established cell lines expressing those CXCR4 mutants, which has a substitution with either with a positive charge residue (e.g. Lys/K or Arg/R) or neutral residue (e.g. Ala/A for Glu/E), a negative charge residue (Asp/D for Glu/E), and systematically analyzed activities of those CXCR4 mutants in respect of their migration, phosphorylation, signaling pathway and immune response to clarify the mechanism underlying WHIM like mutation CXCR4^E343K^ found in a family with WHIM syndrome at molecular level in vitro. Consistent with previous studies, our results show that CXCR4^E343K^ mutation delays the receptor internalization and desensitization [15], considering the simultaneous appearance of WHIM clinical manifestations, by which the authors suggested CXCR4^E343K^ is a WHIM-like mutation. To further investigate downstream events of activated CXCR4 mutants, we detected the phosphorylation of ERK, p38, JNK, PI3K and AKT, and found that their phosphorylation in cells overexpressing CXCR4^E343K^ was lasted longer than that in cells overexpressing CXCR4^WT^ (Figs. 3a-f and 4c-f). Time course test showed that the highest phosphorylation levels of ERK, p38, JNK, PI3K and AKT in cells overexpressing CXCR4^WT^ and in cells overexpressing CXCR4^E343K^ at 5 and 10 min post-treatment with SDF-1 respectively (Figs. 3a-f, 4c-f). These results suggest that the E343K mutation delays the receptor internalization, thus leading delayed and procrastinated phosphorylation of ERK, p38, JNK, PI3K and AKT. To further elucidate the effect of E343K mutation or charge change at 343 site of the receptor tail on the receptor functions, we tested immigration activities and immune responses of all mutants, results show that CXCR4^WT^ and CXCR4^E343D^ had similar migration rate, but CXCR4^E343R^ as well as CXCR4^E343A^ had faster migration activities. Quantification of cytokine mRNA expression showed higher levels and longer expression of TNFa and IL6 were introduced in the CXCR4^E343R^ and CXCR4^E343A^ mutants after activated with SDF1 compared with that in CXCR4^WT^ and CXCR4^E343D^ (Figs. 2, 3 and 4). Consideration of only charge change created in all the CXCR4 mutants, either positive charge (CXCR4^E343R^ and CXCR4^E343k^) or neutral charge (CXCR4^E343A^) replacement at the 343 site of receptor tail, cellular phenotypes of internalization and migration and immune responses are different from CXCR4^WT^ and CXCR4^E343D^, the mutant with no charge change, given us that charge change at the 343site is the unique factor for the introduction of the activity abnormality of the mutants, CXCR4^E343R^, CXCR4^E343k^ and CXCR4^E343A^, demonstrating that the negative charge is essential for mentioning physiological functions of CXCR4, otherwise, pathological events will occur, causing WHIM syndrome.

## Conclusions

We mutated the 343 site residue of glutamic acid (Glu/E) with to negatively charged residue asparitic acid (Asp/D), or positively charged residue lysine (Lys/K) or arginie (Arg/R), or non-charged residue alanine (Ala/A) to determine the signaling properties and functions of the CXCR4. Results from our studies have revealed that negative charge of 343 site is essential for physiologically functional CXCR4, charge-changing of residue at the 343 of receptor tailor dysregulates the down-stream G-protein signal events. This discovery is important for understanding the mechanisms of CXCR4^E343K^ causing WHIM syndrome, expended our understanding of WHIM pathogenesis.

## Methods

### Plasmid construction and transduction of human Hela cells

The full-length CXCR4 (NM_003467.3) was amplified by PCR using primers: 5′-TCTAGAA TGGTGAGCAAGGGCGAGG-3′ and 5′-CTCGAGAATGCTGGAGTGAAAACTTTGAAG-3′, then was subcloned into a lentiviral plasmid pReceiver-Lv201. The mutant CXCR4 was generated by mutagenesis according the manufacturer’s instructions (TAKARA, China).

### Cell culture

HeLa and HEK293T cell lines were obtained from the Institute of Basic Medical Sciences, Chinese Academy of Medical Sciences (Beijing, China). Cells was maintained in MEM medium supplemented with 10% heat-inactivated fetal bovine serum (FBS), 2 mM L-glutamine, 100 U/ ml penicillin and 100 μg/ml streptomycin (Solarbio, China) at 37 °C in a 5% CO2 incubator [33]. The recombinant plasmid pReceiver-Lv201 was transfected into HEK293T cells with lentivirus packaging mix plasmids to pack the expression lentivirus using polyetherimide (PEI) which has been described previously [34]. The recombinant plasmid pReceiver-Lv201 was transfected into HEK293T cells with lentivirus packaging mix plasmids to pack the expression lentivirus using polyetherimide (PEI) which has been described previously. The healing of scratches was observed and photographed using Zeiss AxioScope A1 microscope.

### Antibodies

Anti-ERK1/2 (1:1000), AKT (1:1000), JNK1/2/3 (1:1000) and p38 (1:1000) antibody were purchased from Cell Signaling Techonlogy (Shanghai, China). Anti-PI3K antibody (1:1000) was purchased from Bioworld Technology (Nanjing, China). Anti-phospho-Erk1/2 antibody (1:1000) was purchased from Boster Biological Technology (Wuhan, China). Anti-phospho-JNK1/2 antibody (1:1000) and anti-phospho-PI3K antibody (1:1000) were purchased from Immunoway (Beijing, China). Anti-phospho-p38 antibody and anti-phospho-AKT antibody (1:1000) were purchased from Bioworld Technology (Nanjing, China). The anti-rabbit/mouse secondary antibody (1:1000) were obtained from Boster Biological Technology (Wuhan, China).

### Wound healing assay

The stably overexpressing HeLa cells were seeded into a 24-well plate and cultured in serum-free MEM overnight. When the cell density reached 80–90%, a scratch was introduced to the monolayer with 200 μL pipette tips. HeLa cells were washed for 2–3 times using PBS and incubated by MEM containing 5% FBS and 50 nM chemokine SDF-1 (Peprotech, UK) [35]. The healing of scratches was observed and photographed using Zeiss AxioScope A1 microscope.

### Flow cytometry and receptor internalization

For surface expression and internalization of the receptor analysis, stably transfected HeLa cells grown on six-plate were stimulated with 50 nM SDF-1 for 0 min or 0 min, 40 min at 37 °C. Reactions were terminated by addition of 4% PFA. Cells were stained with Mouse mAb IgG isotype control (Cell Signaling), anti-CXCR4 (4G10) primary antibody (Santa Cruz Biotechnology) and anti-mouse IgG(H + L) conjugate with Alexa Flour 647 (Cell Signaling) and analyzed by using a four-color FACSCalibur flow cytometer equipped with CELLQUEST PRO software (BD LSRFortessa™ X-20) and analyzed using FlowJo software.

### Transwell migration assay

The HeLa cells were seeded into a 24-well plate and cultured in serum-free MEM overnight. 2 × 10^4^ Hela cells were seeded into the upper chambers (consisted of polycarbonate filters, 8 μm pore size) of Transwell (Millipore). Serum-free MEM medium with SDF-1 (50 nM) was added to the bottom chamber. After incubation for 5 h, cells remained on the upper membrane were removed with a cotton swab, while cells that had migrated through the membrane were fixed in formaldehyde, stained with crystal violet and imaged using Zeiss AxioScope A1 microscope, the cell number was quantified using ImageJ software [36].

### Reverse transcription quantitative polymerase chain reaction (RT-qPCR)

Total RNA was extracted from cells using Trizol reagent (TAKARA, Japan) and reverse-transcribed into complementary DNA (cDNA) (Takara, Japan) using 500 ng RNA for qPCR according to the manufacturer’s instructions (Mei5bio, China) The primers used in the qPCR were: IL-6 F: 5′-TGGATGAGCTGAACTGTACCC-3′, IL-6 R: 5′-GCTTGCCAAGGATT GTGAGT; TNF-α F: 5′-CGGAATTCGCCACCATGAGCACTGAAAGC-3′, TNF-α R: 5′-CGGA ATTCGCCACCATGAGCACTGAAAGC-3′; β-actin F: 5′-GATGGAAAGTGACCCGCA-3′, β-actin R: 5′-GAGGAAGACGCAGAGGTTTG-3′.

### Western blot

The stably overexpressing HeLa cells were seeded into 6-well plates and cultured for 24 h, and maintained in 1 ml of serum-free MEM for 6 h at 37 °C before stimulation. After stimulation with SDF-1 (50 nM), cells were collected and washed three times with cold PBS, then total protein was extracted using RIPA lysis buffer (10 mM Tris pH 7.4, 150 mM NaCl, 1% NP-40, 0.5% sodium deoxycholate, 1 mM PMSF, and protease inhibitor). Proteins (30 μg) were separated via SDS-PAGE then transferred onto a nitrocellulose membrane. The membrane was blocked with 5% skim milk powder in Tris-buffered saline (TBS) at room temperature for 1 h. Subsequently, the blots were incubated at 4 °C overnight with primary antibodies against phosphorylated p-ERK1/2, ERK1/2, p-JNK1/2/3, JNK1/2/3, p-p38, p38, p-PI3K, PI3K, p-AKT, AKT. The secondary antibodies were horseradish peroxidase (HRP)-linked anti-rabbit IgG, and HRP-linked anti-mouse IgG. The intensity of the protein bands was analyzed by the ImageJ software.

### Immunofluorescence

The stable cell lines were grown and treated with SDF-1 (50 nM). Cells were fixed with 4% paraformaldehyde for 10 min at room temperature as described [37]. Immunostaining of p65 was done as described above [38]. Secondary, AlexaFluor 647-conjugated donkey anti-goat antibody was used at a concentration of 1:1000 (Thermofisher, USA) for 90 min. Nuclei were counterstained with 4 V,6-diamidino-2-phenylindole (DAPI, sigma). Images of fixed cells were taken with a Zeiss LSM710 Microscope with a 63 X 1.4 DIC Plan-Apochromat oil-immersion objective.

### Statistical analysis

All experiments were replicated at least three times. The experiments results were presented as the mean ± standard deviation. A one-way analysis of variance (ANOVA) was utilized to calculate *P*-values via GraphPad Prism 7.0 software. The value *P* < 0.05 was considered as statistically significant.

## Supplementary Information

**Additional file 1: Fig. S1** Ligand-induced internalization of wild type and mutant CXCR4. The mean levels of cell surface CXCR4 at the 40 min post-stimulation with SDF-1was examined by flow cytometry from 3 independent experiments. *p < 0 .05, **p <0 .01, ***p <0 .001 compared to the WT group.

## Data Availability

All data generated and analyzed for this study are included in this published article.

## Abbreviations

WHIM: Warts, hypogammaglobulinemia, recurrent bacterial infections and myelokathexis
PID: syndrome is a primary immunodeficiency disease
HPV: papillomavirus
CXCR4: cysteine-X -cysteine chemokine receptor 4
PKC: protein kinase C
GPCR: G-protein-coupled receptor.

## References

[1] Dotta L, Tassone L, Badolato R (2011). Clinical and genetic features of warts, Hypogammaglobulinemia, infections and Myelokathexis (WHIM) syndrome. Curr Mol Med.

[2] Beaussant Cohen S, Fenneteau O, Plouvier E, Rohrlich PS, Daltroff G, Plantier I, Dupuy A, Kerob D, Beaupain B, Bordigoni P (2012). Description and outcome of a cohort of 8 patients with WHIM syndrome from the French severe chronic neutropenia registry. Orphanet J Rare Dis.

[3] Galli J, Pinelli L, Micheletti S, Palumbo G, Notarangelo LD, Lougaris V, Dotta L, Fazzi E, Badolato R (2019). Cerebellar involvement in warts Hypogammaglobulinemia immunodeficiency myelokathexis patients: neuroimaging and clinical findings. Orphanet J Rare Dis.

[4] Liu Q, Pan C, Lopez L, Gao J, Velez D, Anaya-O'Brien S, Ulrick J, Littel P, Corns JS, Ellenburg DT (2016). WHIM syndrome caused by Waldenstrom's Macroglobulinemia-associated mutation CXCR4 (L329fs). J Clin Immunol.

[5] Majumdar S, Murphy PM. Adaptive Immunodeficiency in WHIM Syndrome. Int J Mol Sci. 2018;20(1). 10.3390/ijms20010003 PMC6337672 30577453

[6] Gorlin RJ, Gelb B, Diaz GA, Lofsness KG, Pittelkow MR, Fenyk JR (2000). WHIM syndrome, an autosomal dominant disorder: clinical, hematological, and molecular studies. Am J Med Genet.

[7] Badolato R, Dotta L, Tassone L, Amendola G, Porta F, Locatelli F, Notarangelo LD, Bertrand Y, Bachelerie F, Donadieu J (2012). Tetralogy of fallot is an uncommon manifestation of warts, hypogammaglobulinemia, infections, and myelokathexis syndrome. J Pediatr.

[8] Zuelzer WW (1964). "Myelokathexis"--a new form of chronic granulocytopenia. Report of a case. N Engl J Med.

[9] Tassone L, Moratto D, Vermi W, De Francesco M, Notarangelo LD, Porta F, Lougaris V, Facchetti F, Plebani A, Badolato R (2010). Defect of plasmacytoid dendritic cells in warts, hypogammaglobulinemia, infections, myelokathexis (WHIM) syndrome patients. Blood.

[10] Haribabu B, Richardson RM, Fisher I, Sozzani S, Peiper SC, Horuk R, Ali H, Snyderman R (1997). Regulation of human chemokine receptors CXCR4. Role of phosphorylation in desensitization and internalization. J Biol Chem.

[11] Busillo JM, Armando S, Sengupta R, Meucci O, Bouvier M, Benovic JL (2010). Site-specific phosphorylation of CXCR4 is dynamically regulated by multiple kinases and results in differential modulation of CXCR4 signaling. J Biol Chem.

[12] Woerner BM, Warrington NM, Kung AL, Perry A, Rubin JB (2005). Widespread CXCR4 activation in astrocytomas revealed by phospho-CXCR4-specific antibodies. Cancer Res.

[13] Heusinkveld LE, Majumdar S, Gao JL, McDermott DH, Murphy PM (2019). WHIM syndrome: from pathogenesis towards personalized medicine and cure. J Clin Immunol.

[14] McDermott DH, Murphy PM (2019). WHIM syndrome: Immunopathogenesis, treatment and cure strategies. Immunol Rev.

[15] Liu Q, Chen H, Ojode T, Gao X, Anaya-O'Brien S, Turner NA, Ulrick J, DeCastro R, Kelly C, Cardones AR (2012). WHIM syndrome caused by a single amino acid substitution in the carboxy-tail of chemokine receptor CXCR4. Blood.

[16] Gao D, Sun H, Zhu J, Tang Y, Li S (2018). CXCL12 induces migration of Schwann cells via p38 MAPK and autocrine of CXCL12 by the CXCR4 receptor. Int J Clin Exp Pathol.

[17] Lu Y, Xing J, Yin X, Zhu X, Yang A, Luo J, Gou J, Dong S, Xu J, Hou T (2019). Bone marrow-derived CD44(+) cells migrate to tissue-engineered constructs via SDF-1/CXCR4-JNK pathway and aid bone repair. Stem Cells Int.

[18] Li F, Xue ZY, Yuan Y, Huang SS, Fan YH, Zhu X, Wei L (2018). Upregulation of CXCR4 through promoter demethylation contributes to inflammatory hyperalgesia in rats. CNS Neurosci Ther.

[19] Isles HM, Herman KD, Robertson AL, Loynes CA, Prince LR, Elks PM, Renshaw SA (2019). The CXCL12/CXCR4 signaling Axis retains neutrophils at inflammatory sites in Zebrafish. Front Immunol.

[20] Yu X, Wang D, Wang X, Sun S, Zhang Y, Wang S, Miao R, Xu X, Qu X (2019). CXCL12/CXCR4 promotes inflammation-driven colorectal cancer progression through activation of RhoA signaling by sponging miR-133a-3p. J Exp Clin Cancer Res.

[21] Tian X, Xie G, Xiao H, Ding F, Bao W, Zhang M (2019). CXCR4 knockdown prevents inflammatory cytokine expression in macrophages by suppressing activation of MAPK and NF-kappaB signaling pathways. Cell Biosci.

[22] Abdelhamid G, El-Kadi AO (2015). Buthionine sulfoximine, an inhibitor of glutathione biosynthesis, induces expression of soluble epoxide hydrolase and markers of cellular hypertrophy in a rat cardiomyoblast cell line: roles of the NF-kappaB and MAPK signaling pathways. Free Radic Biol Med.

[23] Peri F, Piazza M, Calabrese V, Damore G, Cighetti R (2010). Exploring the LPS/TLR4 signal pathway with small molecules. Biochem Soc Trans.

[24] Kawai T, Choi U, Cardwell L, DeRavin SS, Naumann N, Whiting-Theobald NL, Linton GF, Moon J, Murphy PM, Malech HL (2007). WHIM syndrome myelokathexis reproduced in the NOD/SCID mouse xenotransplant model engrafted with healthy human stem cells transduced with C-terminus-truncated CXCR4. Blood.

[25] Aghamohammadi A, Abolhassani H, Puchalka J, Greif-Kohistani N, Zoghi S, Klein C, Rezaei N (2017). Preference of genetic diagnosis of CXCR4 mutation compared with clinical diagnosis of WHIM syndrome. J Clin Immunol.

[26] Heusinkveld LE, Yim E, Yang A, Azani AB, Liu Q, Gao JL, McDermott DH, Murphy PM (2017). Pathogenesis, diagnosis and therapeutic strategies in WHIM syndrome immunodeficiency. Expert Opin Orphan Drugs.

[27] Evans MO, McDermott DH, Murphy PM, Petersen MM (2019). Abnormal newborn screen in a WHIM syndrome infant. J Clin Immunol.

[28] McDermott DH, Murphy PM (2019). Plerixafor for the treatment of WHIM syndrome. Reply N Engl J Med.

[29] Cronshaw DG, Nie Y, Waite J, Zou YR (2010). An essential role of the cytoplasmic tail of CXCR4 in G-protein signaling and organogenesis. PLoS One.

[30] Orsini MJ, Parent JL, Mundell SJ, Marchese A, Benovic JL (1999). Trafficking of the HIV coreceptor CXCR4. Role of arrestins and identification of residues in the c-terminal tail that mediate receptor internalization. J Biol Chem.

[31] Balabanian K, Levoye A, Klemm L, Lagane B, Hermine O, Harriague J, Baleux F, Arenzana-Seisdedos F, Bachelerie F (2008). Leukocyte analysis from WHIM syndrome patients reveals a pivotal role for GRK3 in CXCR4 signaling. J Clin Invest.

[32] Mueller W, Schutz D, Nagel F, Schulz S, Stumm R (2013). Hierarchical organization of multi-site phosphorylation at the CXCR4 C terminus. PLoS One.

[33] Zhao Q, Wang W, Cui J (2019). Melatonin enhances TNF-alpha-mediated cervical cancer HeLa cells death via suppressing CaMKII/Parkin/mitophagy axis. Cancer Cell Int.

[34] Kanthamneni N, Yung B, Lee RJ (2016). Effect of Eudragit on in vitro transfection efficiency of PEI-DNA complexes. Anticancer Res.

[35] Liu SB, Wang HF, Xie QP, Li G, Zhou LB, Hu B (2020). LncRNA SNHG16 promotes migration and invasion through suppression of CDKN1A in clear cell renal cell carcinoma. Eur Rev Med Pharmacol Sci.

[36] Liu T, Ma Q, Zhang Y, Wang X, Xu K, Yan K, Dong W, Fan Q, Zhang Y, Qiu X (2019). Self-seeding circulating tumor cells promote the proliferation and metastasis of human osteosarcoma by upregulating interleukin-8. Cell Death Dis.

[37] Rubin JB, Kung AL, Klein RS, Chan JA, Sun Y, Schmidt K, Kieran MW, Luster AD, Segal RA (2003). A small-molecule antagonist of CXCR4 inhibits intracranial growth of primary brain tumors. Proc Natl Acad Sci U S A.

[38] Guo W, Imai S, Yang JL, Zou S, Li H, Xu H, Moudgil KD, Dubner R, Wei F, Ren K (2018). NF-KappaB pathway is involved in bone marrow stromal cell-produced pain relief. Front Integr Neurosci.

