# Supplementary figures and images for: The negative charge of the 343 site is essential for maintaining physiological functions of CXCR4
Source: BMC Mol Cell Biol. 2021 Jan 23;22:8. doi: 10.1186/s12860-021-00347-9 (PMC7825245; doi:10.1186/s12860-021-00347-9)

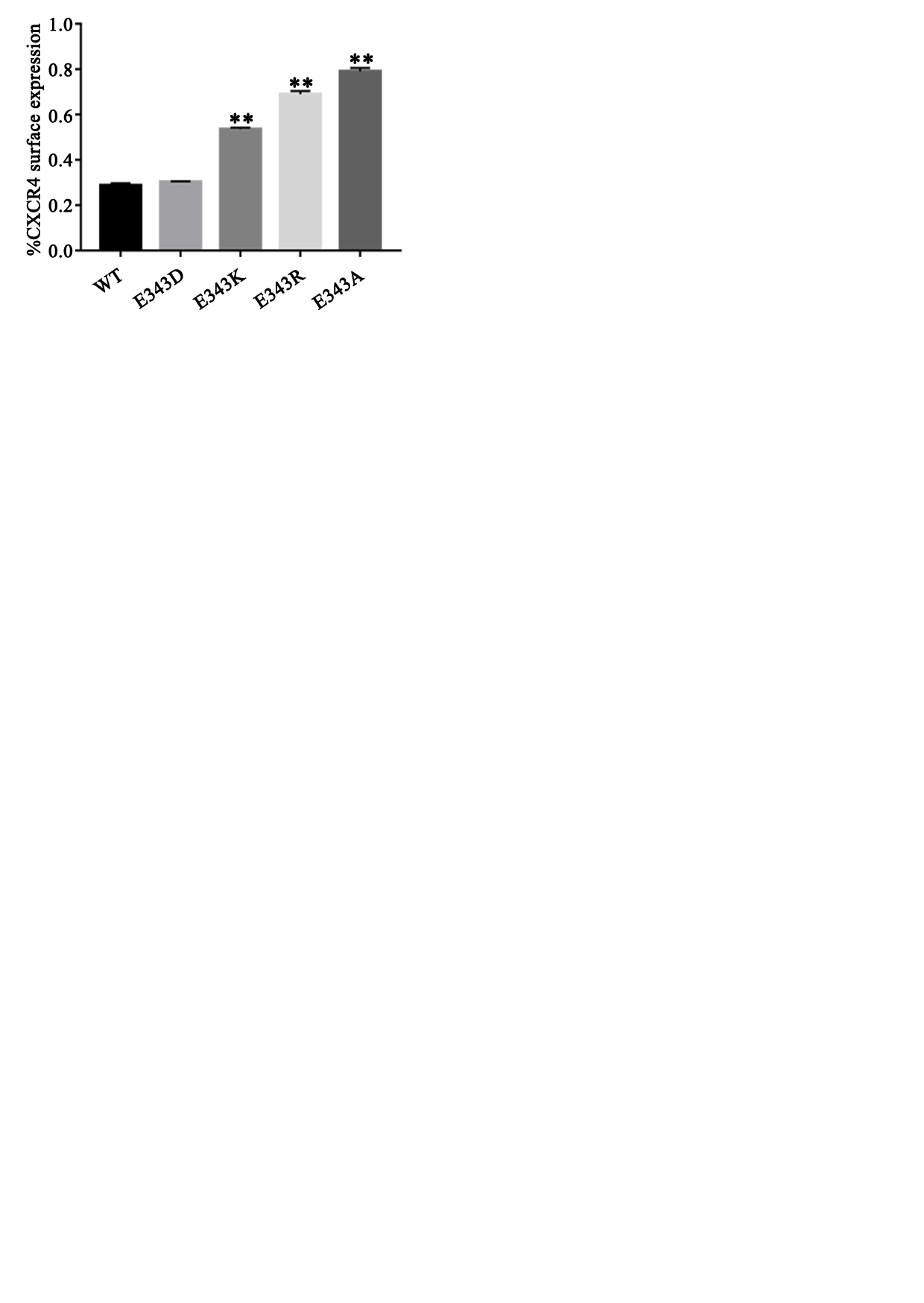

Supplement: Supplementary file 1 — Additional file 1: Fig. S1 Ligand-induced internalization of wild type and mutant CXCR4. The mean levels of cell surface CXCR4 at the 40 min post-stimulation with SDF-1was examined by flow cytometry from 3 independent experiments. *p < 0 .05, **p <0 .01, ***p <0 .001 compared to the WT group. [file 12860_2021_347_MOESM1_ESM.zip › 12860_2021_347_MOESM1_ESM/Figure S1.tif]
